# Supplementary material for: Chrysoviruses Inhabited Symbiotic Fungi of Lichens
Source: Viruses. 2019 Dec 3;11(12):1120. doi: 10.3390/v11121120 (PMC6949994; doi:10.3390/v11121120)
Supplement: Supplementary file 1 [file viruses-11-01120-s001.pdf]

viruses

Supplementary material

primer	5´-3´sequence	template	position	reference
oligo(dT)	GGCCACGCGTCGACTAGTACTCGAGTTTTTTTTTTTTTTTTT		3´poly(A) tail	
tagged random	CGATCGATCATGATGCAATGCNNNNNN			Darissa et al. 2010
single specific	CGATCGATCATGATGCAATGC			Darissa et al. 2010
2381	CATGTTCTGTATCACTAACC	RNA1	448r	
2382	CCATACTTGTAGGCTAAATGTG	RNA1	407r	
647	GGCAGGAAGATACCAAGAGTTTATACAG	RNA1	3165f	
876	CAGTCAGTAAGGAAGAATTAGCGCA	RNA1	3215f	
554	AACCTATACACATCTTATCTTCAGCCAA	RNA2	478r	
715	CATAGTACATTTGGTAACCTATACACATC	RNA2	493r	
618	AGACAGTATATCAGGAAATGCCGAC	RNA2	2850f	
875	AAGTGAGCGACCAGTTAACCAAG	RNA2	2907f	
2298	AGCCATTTCAGTCTTTGTAAATTGTGG	RNA3	320r	
619	CCTATATTTCTTCTCCTGCTTACC	RNA3	287r	
620	CTGTGGTGGCTTAGGTTTAATGG	RNA3	2450f	
2616	AAAGTTGAGCGAAAGACAC	RNA3	2516f	
566	TCCTCTACACAATCAATCACCTCT	RNA4	263r	
568	TGATGCTGGCTGATAAAGAAAC	RNA4	2435f	
648	TTACAATGATGGAACGCAAGATCAG	RNA4	2472f	
846	GTTGTATGGGGTAGTGGAACAAAGGATAAACGGTT	RNA4	2017f	
847	TCGTATCACCTGCCCCTATCCTACCGTG	RNA4	2338r	
2352	AAGCATGCATTCATTAATAAGGA	RNA1	1365f	
2340	GAATAGCCTGATCAAGATCTG	RNA1	1606r	
1901	TCGTCGCCGTAAACGACCCC	GAPDH		
1902	CGCCCTTGAACTGGCCGTGT	GAPDH		
ITS1	TCCGTAGGTGAACCTGCGG	ITS		White et al. 1990
ITS4	TCCTCCGCTTATTGATATGC	ITS		White et al. 1990
MY1574	Cy3-TCCTCGTTGAAGAGC	universal fungi		Baschien et al. 2008
Cy3-859	Cy3-GGGCAAATAGAGAGAAGG	RNA3		

Supplementary Table 1. Primers and probes used in this work

virus	acronym	AC RNA1	AC RNA2	AC RNA3	AC RNA4
Alternaria alternata chrysovirus		YP_009553287	QBA82441		
Amasya cherry disease associated chrysovirus	ACDAC	YP_001531163	NC_009946		YP_001531161
Anturium mosaic-associated chrysovirus		ACU11563	FJ899676		
Aspergillus fumigatus chrysovirus	AfuCV	FN178512	CAX48751	YP_009508103	YP_009508106
Beauveria bassiana chrysovirus 1	BbCV1			AZT88573	AZT88574
Bipolaris maydis chrysovirus	BmCV1				ARM36037
Botryosphaeria dothidea chrysovirus		AJD14830	AJD14831		
Brassica campestris chrysovirus	BrcCV1	AKU48197	KP782030		
Colletotrichum fructicola chrysovirus 1		YP_009551629	YP_009551635		
Colletotrichum gloeosporioides chrysovirus 1	CgCV1	ALW95408	ALW95409		ALW95410
Cryphonectria nitschkei chrysovirus 1	CnCV1	ACT79255	ACT79251		ABI20757
Fusarium oxysporum f.sp. dianthi mycovirus 1		YP_009158913			
Grapevine chrysovirus	GrCV1				AFX73020
Helminthosporium victoriae virus 145S	HvV145S	AF297176	AF297177		
Chrysothrix chrysovirus 1	CcCV1	MN625832	MN625833	MN625834	MN625835
Isaria javanica chrysovirus	IjCV	YP_009337840	APR73429	YP_009337890	YP_009337841
Lepraria chrysovirus 1	LiCV1	MN393162	MN393161		MN393163
Macrophomina phaseolina chrysovirus	MpCHrV1	ALD89090	ALD89091	ALD89092	ALD89093
Penicillium chrysogenum virus	PcV	AF296439	YP_392483	YP_392484	YP_392485
Penicillium italicum chrysovirus			QCZ35877		
Penicillium raistrickii chrysovirus 1	PrCV1	AZT88567	AZT88568	AZT88569	AZT88570
Penicillium roseopurpureum chrysovirus	ProCV	AYP71812	AYP71813	AYP71814	AYP71815
Persea americana chrysovirus		AJA37498	AJA37499		
Raphanus sativus chrysovirus	RasCV1	AFE83590	AFE83591		
Verticillium dahliae chrysovirus 1	VdCV1	ADG21213	ADG21214		YP_009507947

Supplementary Table 2. Viruses and sequences used in this paper

	PrCV1	PcV	ProCV	AfuCV	BbCV1	IjCV	CcCV1	MpCHrV1
PrCV1		60	54	34	23	23	23	17
PcV	60		52	34	23	23	23	17
ProCV	54	52		34	23	22	23	16
AfuCV	34	34	34		21	22	24	20
BbCV1	23	23	23	21		52	22	19
IjCV	23	23	22	22	52		22	19
CcCV1	23	23	23	24	22	22		17
MpCHrV1	17	17	16	20	19	19	17	

Supplementary Figure 1. Amino acid sequence identity and heat map of protein encoded on RNA3 segment.

	CnCV1	VdCV1	PrCV1	PcV	ProCV	AfuCV	CcCV1	BbCV1	IjCV	GrCV	MpCHrV1	BmCV1	ACDACV	CgCV1
CnCV1		55	28	27	27	29	28	27	29	30	31	23	21	22
VdCV1	55		30	30	29	30	29	30	30	32	34	25	21	25
PrCV1	28	30		88	82	70	44	41	41	43	37	24	22	24
PcV	27	30	88		81	69	43	41	40	43	37	24	22	23
ProCV	27	29	82	81		69	43	40	40	43	37	24	22	23
AfuCV	29	30	70	69	69		45	41	40	44	39	24	22	23
CcCV1	28	29	44	43	43	45		40	40	43	39	24	25	23
BbCV1	27	30	41	41	40	41	40		74	49	36	25	21	22
IjCV	29	30	41	40	40	40	40	74		49	36	25	22	23
GrCV	30	32	43	43	43	44	43	49	49		41	27	24	25
MpCHrV1	31	34	37	37	37	39	39	36	36	41		25	23	24
BmCV1	23	25	24	24	24	24	24	25	25	27	25		31	28
ACDACV	21	21	22	22	22	22	25	21	22	24	23	31		25
CgCV1	22	25	24	23	23	23	23	22	23	25	24	28	25	

Supplementary Figure 2. Amino acid sequence identity and heat map of protein encoded by RNA4 segment.

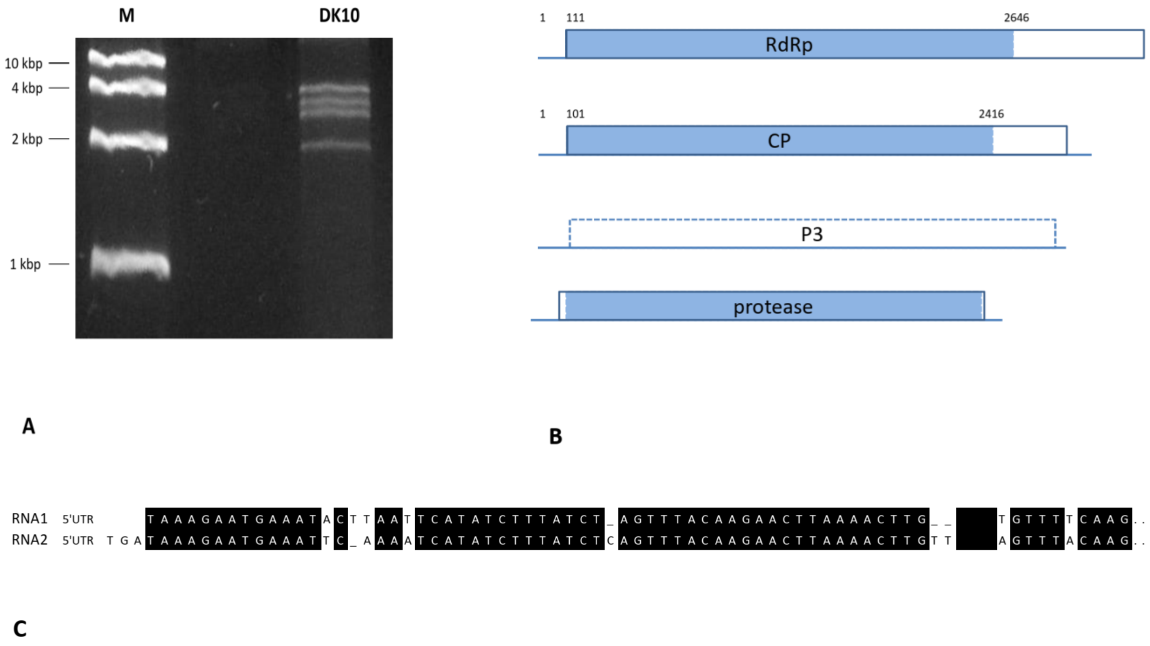

Supplementary Figure 3. (A) Agarose gel separation of dsRNA extracted from DK10 lichen sample. Marker - 10 kbp, 4 kbp, 2 kbp, 1 kbp. (B) Sequenced regions of LiCV1 chrysovirus from DK10 sample. Blue rectangles represent position of sequenced regions. (C) Alignment of 5′UTR sequences of RNA1 and RNA2 segments of LiCV1.
